# Evaluation of Systems’ Irregularity and Complexity: Sample Entropy, Its Derivatives, and Their Applications across Scales and Disciplines

**DOI:** 10.3390/e20100794

**Published:** 2018-10-16

**Authors:** Anne Humeau-Heurtier

**Affiliations:** Laboratoire Angevin de Recherche en Ingénierie des Systèmes (LARIS), University of Angers, 62 avenue Notre-Dame du Lac, 49000 Angers, France; anne.heurtier@univ-angers.fr

**Keywords:** sample entropy, multiscale entropy, fuzzy entropy, multivariate data

Based on information theory, a number of entropy measures have been proposed since the 1990s to assess systems’ irregularity, such as approximate entropy, sample entropy, permutation entropy, intrinsic mode entropy, and dispersion entropy to cite only a few. Among them, sample entropy has been used in a very large variety of disciplines for both univariate and multivariate data. However, improvements to the sample entropy algorithm are still being proposed because sample entropy is unstable for short time series, may be sensitive to parameter values, and can be too time-consuming for long data.

At the same time, it is worth noting that sample entropy does not take into account the multiple temporal scales inherent in complex systems. It is maximized for completely random processes and is used only to quantify the irregularity of signals on a single scale. This is why analyses of irregularity—with sample entropy or its derivatives—at multiple time scales have been proposed to assess systems’ complexity.

This Special Issue invited contributions related to new and original research based on the use of sample entropy or its derivatives. The papers published in this Special Issue can be divided into two categories. First, some papers present new applications of sample entropy or its derivatives. Second, some papers propose improvements to sample entropy or its derivatives. 

In addition to these articles, Sun et al. [1] performed a systematic review to summarize the complexity differences of important signals in patients with asthma. They obtained the overall trend of entropy change in the physiological signals related to asthma, evaluated the potential of using entropy of biological dynamics as new clinical indices, and discussed possible strategies for future research. The authors mention that entropy of heart rate variability (HRV), airflow, center of pressure, and respiratory system impedance are lower in patients than in healthy people, whereas the entropies of respiratory sound, airway resistance, and reactance are higher in patients than in healthy people. This might be explained by the unstableness of local respiratory tract and the increase in breathing difficulty.

## 1. Applications of Sample Entropy or Its Derivative

In the biomedical field, Liao et al. used multiscale entropy to study complexity of skin blood flow in type 2 diabetic patients with peripheral neuropathy and in healthy controls [2]. Skin blood flow was recorded at the first metatarsal head using laser Doppler flowmetry in response to locally applied pressure and heating. Indeed, quantification of skin blood flow responses to loading pressure and thermal stresses may be a reasonable way to assess the risk of diabetic foot ulcer. Multiscale entropy was computed using a modified sample entropy algorithm. The results showed that during reactive hyperemia and the biphasic response induced by local heating, the modified sample entropy in diabetics presents only small changes compared to baseline but undergoes significant changes in controls. Moreover, during baseline and skin blood flow responses—except for the pressure loading period—the modified sample entropy at small scales exhibits different transitions between the two groups. The findings support the use of nonlinear measures of skin blood flow responses induced by mechanical and thermal stresses to assess the risk of diabetic foot ulcer.

Simons et al. studied electroencephalogram (EEG) of Alzheimer’s disease (AD) and age-matched control subjects [3]. For this purpose, they used fuzzy entropy. Their results showed that fuzzy entropy is lower in Alzheimer’s patients than in control subjects for electrodes T6, P3, P4, O1, and O2. Moreover, fuzzy entropy led to better results than approximate entropy or sample entropy when diagnostic accuracy was computed with the receiver operating characteristic curves. However, the authors note that the results are dependent on the input parameters used in the fuzzy entropy computation. 

Ruiz-Gomez et al. aimed to detect AD and its prodromal form (i.e., mild cognitive impairment, MCI) from healthy controls through an EEG-based methodology [4]. For this purpose, after a data preprocessing step, the authors used a combination of spectral measures and nonlinear methods, with both frequency (spectral features) and time domain (nonlinear features: Lempel–Ziv complexity, central tendency measure, sample entropy, fuzzy entropy, and auto mutual information) analyses applied to EEG recordings. Furthermore, to avoid redundant features sharing similar information, an automatic feature selection stage based on the fast correlation-based filter (FCBF) was used. Finally, different multiclass classifiers were tested: logistic discriminant analysis (LDA), quadratic discriminant analysis (QDA), and multilayer perceptron neural network (MLP). The results showed that both AD and MCI elicit changes in the EEG background activity, including a slowing of EEG rhythms, alterations in the frequency distribution of the power spectrum, a complexity loss, a regularity increase, and a variability decrease. Spectral and nonlinear features allowed the brain abnormalities associated with AD and MCI to be characterized. Moreover, the brain activity in AD patients is less complex, more regular, and less variable than in MCI and healthy control subjects.

In their work, Kumar et al. proposed a method for an automatic diagnosis of myocardial infarction (MI) using ECG beat with a flexible analytic wavelet transform (FAWT) method [5]. For this purpose, using lead-2 ECG signals, first, a preprocessing step was performed to remove the baseline wandering and other noise present in the ECG signals. Then, ECG signals were segmented into the beats. Furthermore, these beats were decomposed up to the 24th level of decomposition using FAWT. Sample entropy was computed from each sub-band signal, which was reconstructed from the corresponding coefficients of the FAWT-based decomposition. The computed features were subjected to random forest (RF), J48 decision tree, back propagation neural network (BPNN), and least-squares support vector machines (LS-SVM) classifiers to separate the ECG beats of MI and normal classes. The authors found that lower frequency sub-band signals show higher sample entropy values for normal ECG beats than MI ECG beats and that higher frequency sub-band signals extracted from normal ECG beats have lower values of sample entropy. Finally, the method used by the authors achieved 99.31% accuracy using LS-SVM classifier with radial basis function kernel.

Fazan et al.’s work is related to the analysis of the changes in physiological complexity entailed by physical training [6]. This work was performed through the study of HRV time series recorded in rats divided into two groups: rats that performed medium intensity training and a sedentary group. HRV signals were recorded five days after the experimental protocol. The analysis of the HRV time series was done with different algorithms: multiscale entropy, multiscale dispersion entropy, and multiscale SDiffq. Multiscale SDiffq is a measure of entropic differences, and differences of entropy between time series and its uncorrelated version, i.e., surrogate data, are used to represent the complexity. From the latter, three quantities (*q*-attributes) were computed: SDiffqmax, *q*max, and *q*zero (SDiffqmax represents the maximum value for SDiffq in the range of *q*, whereas *q*max and *q*zero represent the *q*-value where SDiffq finds its maximum and zero values, respectively). The results showed that the significant difference was only found between trained and sedentary rats in the mean *q*max at long time scales. Physical training therefore increased the system complexity. The authors also conclude that multiscale SDiffq is an alternative tool for characterizing the complexity of HRV time series as it can add information in some situations where multiscale entropy is not accurate enough.

Shi et al. studied the within-subject changes of HRV during walking with a regular speed of 5 km/h on a treadmill (the Walk protocol) compared to those in a resting seated position (the Rest protocol) [7]. The analysis of the time series was performed with eight different entropy measures: approximate entropy, corrected approximate entropy, sample entropy, fuzzy entropy without removing local trend, fuzzy entropy with local trend removal, permutation entropy, conditional entropy, and distribution entropy. The authors also explored the potential effects of nonstationary linear or very low-frequency trend. From their results, the authors note the importance of stating whether detrending has been performed in studies and, if so, which process has been performed.

Bakhchina et al. were interested in the “mind–body” relationship and explored how the system organization of behavior could be reflected in the irregularity of the heart rate, as measured by sample entropy [8]. Thus, they proposed a model explaining HRV in relation to neuronal processes in the brain. For this, they studied the sample entropy of the heart rate in different conditions. The results revealed that irregularity of the heart rate reflect the properties of a set of functional systems subserving current behavior, with higher irregularity corresponding to later-acquired and more complex behavior. This showed that the dynamics of functional systems supporting current behavior is reflected not only in activity of the brain but also in the activity of the rest of the body. The authors finally conclude that sample entropy of the heart rate can be used as a new tool to study psychological processes and organization of behavior. 

Ye et al. proposed a method for the recognition of driving fatigue [9]. They used sample entropy associated with kernel principal component analysis to recognize driving fatigue. The framework was tested on EEG data. Using support vector machine for classification, a driving fatigue state recognition model was constructed. The results were compared with the ones given by sample entropy alone, fuzzy entropy, and combination entropy. The authors showed that their approach significantly improved the classification recognition rate compared with the traditional sample entropy, fuzzy entropy, and combination entropy. Moreover, fuzzy entropy and combination entropy associated with kernel principal component analysis give worse results than those obtained with sample entropy and kernel principal component analysis. 

In another field of application, Lin et al. [10] proposed a structural health monitoring (SHM) system based on multiscale cross-sample entropy (MSCE) to detect damage locations in multibay three-dimensional structures. Through MSCE, the degree of dissimilarity between the response signals of vertically adjacent floors was used to localize damage for each bay analysis. Moreover, a damage index was proposed for rapidly and efficiently diagnosing the damaged floor, axis, and bay in the structure. The work of the authors shows the feasibility and further potential of the proposed SHM system for the detection and localization of damage in large and complex structures.

In a completely different context, Yin et al. analyzed the complexity of carbon market and an illustration was performed on pilot carbon markets in China [11]. Because of the short length of the time series used, the analysis was done through the use of the modified multiscale entropy proposed by Wu et al. in 2013. The results showed an overall low complexity in those carbon markets, far smaller than that in the European carbon market. Furthermore, the complexity of the carbon market (except Chongqing) was found higher in small time scales than in large scales. Moreover, complexity level in most pilot markets increased as the markets developed, showing an improvement in market efficiency. 

## 2. Improvements of Sample Entropy or Its Derivatives

Sample entropy has the drawback of necessitating a long computational time. This is why Manis et al. presented three algorithms to compute sample entropy quickly [12]. The first algorithm is an extension of the kd-trees algorithm, customized for sample entropy. The second one is an extension of an algorithm initially proposed for approximate entropy—the bucket-assisted algorithm—customized for sample entropy. The last one is the most rapid for specific values of *m*, *r*, time series length, and signal characteristics.

Looney et al. proposed an analysis dealing with multivariate sample entropy [13]. The authors first revisited the embedding of multivariate delay vectors. They also proposed a new multivariate sample entropy algorithm. Their results showed the improved performance of this new algorithm over existing work for synthetic data and for classifying wake and sleep states from real-world physiological data. Moreover, they showed that synchronized regularity dynamics are uniquely identified via the new multivariate sample entropy analysis.

Chen et al. proposed the hierarchical cosine similarity entropy (HCSE) to overcome some limitations of the multiscale sample entropy, such as undefined entropy value for short time series [14]. HCSE takes both lower and higher frequency components into consideration. The algorithm proposed by the authors is composed of three steps. First, the hierarchical decomposition is used to decompose the time series under study into subsequences. Second, the sample entropy is modified using Shannon entropy rather than conditional entropy. Moreover, angular distance is used instead of Chebyshev distance. Third, the complexity of each subsequence is quantified by the modified sample entropy. An application of HCSE is shown first on synthetic signals and then on the classification accuracy of real ship-radiated noise, which is the main signal source of passive sonar for underwater target detection and recognition. The results show the superiority of the new algorithm over traditional multiscale entropy. 

Azami et al. proposed to assess the impact of coarse-graining in multiscale entropy estimations based on both sample entropy and dispersion entropy [15]. Thus, the computation of multiscale entropy relies on two steps: (i) a coarse-graining approach, which is a combination of moving average filter and downsampling process; (ii) computation of the sample entropy for each scale factor, i.e., for each coarse-grained time series. A low-pass Butterworth filter was proposed as an alternative to moving average. Thus, the authors compared existing and newly proposed coarse-graining approaches for univariate multiscale entropy estimation. Among others, their results show that the downsampling may lead to increased or decreased values of entropy depending on the sampling frequency of the time series. The authors also concluded that downsampling within the coarse-graining procedure may not be needed to quantify the complexity of signals, especially for short ones. Moreover, the authors showed that dispersion entropy leads to more stable results than sample entropy in the estimations based on coefficient of variation values and ensures that the entropy values are defined at all temporal scales.

Fuzzy entropy is a derivative of sample entropy that has shown to give better results than sample entropy in some situations; fuzzy entropy presents a stronger relative consistency and shows less dependence on data length than sample entropy. However, fuzzy entropy still has some drawbacks as it depends on the number of samples in the data under study: The shorter the signal, the lower is the precision of the fuzzy entropy values. This is why Girault et al. proposed a new fuzzy entropy measure that presents better precision than the standard fuzzy entropy [16]. This is performed by increasing the number of samples used in the computation of the entropy measure without changing the length of the time series. For this purpose, the constraint of the mean value in the comparison of the patterns is removed. Moreover, not only translated patterns, but reflected, inversed, and glide-reflected patterns are also considered. The new measure (so-called centered and averaged fuzzy entropy) was applied to synthetic and biomedical signals (fetal heart rate time series). The results showed that the centered and averaged fuzzy entropy leads to more precise results than the standard fuzzy entropy does.
